# A new (Ba, Ca) (Ti, Zr)O_3_ based multiferroic composite with large magnetoelectric effect

**DOI:** 10.1038/srep32164

**Published:** 2016-08-24

**Authors:** M. Naveed-Ul-Haq, Vladimir V. Shvartsman, Soma Salamon, Heiko Wende, Harsh Trivedi, Arif Mumtaz, Doru C. Lupascu

**Affiliations:** 1 Institute for Materials Science and Center for Nanointegration Duisburg-Essen (CENIDE), University of Duisburg-Essen, Universitätsstraße 15, 45141 Essen, Germany; 2 Faculty of Physics and Center for Nanointegration Duisburg-Essen (CENIDE), University of Duisburg-Essen, Lotharstraße 1, 47057 Duisburg, Germany; 3 Department of Physics, Quaid-i-Azam University, Islamabad 45320, Pakistan

## Abstract

The lead-free ferroelectric 0.5Ba(Zr_0.2_Ti_0.8_)O_3_ − 0.5(Ba_0.7_Ca_0.3_)TiO_3_ (BCZT) is a promising component for multifunctional multiferroics due to its excellent room temperature piezoelectric properties. Having a composition close to the polymorphic phase boundary between the orthorhombic and tetragonal phases, it deserves a case study for analysis of its potential for modern electronics applications. To obtain magnetoelectric coupling, the piezoelectric phase needs to be combined with a suitable magnetostrictive phase. In the current article, we report on the synthesis, dielectric, magnetic, and magnetoelectric characterization of a new magnetoelectric multiferroic composite consisting of BCZT as a piezoelectric phase and CoFe_2_O_4_ (CFO) as the magnetostrictive phase. We found that this material is multiferroic at room temperature and manifests a magnetoelectric effect larger than that of BaTiO_3_ −CoFe_2_O_4_ bulk composites with similar content of the ferrite phase.

A material is usually called functional from an application perspective, when it has one or more properties that are sensitive to environmental variables such as pressure, temperature, electric, or magnetic fields. Functional materials utilize native properties to achieve an intelligent action^1^. Among these functional materials, multiferroic materials have attracted very high interest^2^,^3^,^4^ due to their magnetoelectric (ME) property, namely the ability to control polarization by a magnetic field and magnetization by an electric field^5^. This feature makes them excellent candidates for electrically written and magnetically read memory technologies^6^,^7^, ambient sensors of magnetic field^8^,^9^, energy harvesting devices^10^, electrical field tunable devices^11^ and current/voltage converters^12^. During the past decade, the search for multiferroics with large magnetoelectric effect has become a prime focus of attention of physicists and material scientists. The scarcity of single phase multiferroics^3^ and their relatively weak magnetoelectric coupling have motivated the search for composite materials.

Typically, a multiferroic composite consists of a ferroelectric (for its piezoelectric property) and a ferro- or ferrimagnet (for its magnetostrictive property). The magnetoelectric effect is achieved via mechanical strain transfer at the interfaces between the two phases. The largest values of the ME effect have been achieved in composites containing excellent piezoelectrics like Pb(Zr_1−*x*_Ti_*x*_)O_3_^13^ or (1−*x*)Pb(Mg_1/3_Nb_2/3_)O_3_-*x*PbTiO_3_^14^. However, restrictions on using lead in electronic equipment^15^ have motivated search for lead-free alternatives. A typical choice is BaTiO_3_ that has a piezoelectric coefficient, *d*_33_, of about 191 pC/N^16^. The values of the direct magnetoelectric coefficients, *α*_*E*_, for BaTiO_3_-based bulk particulate composites are typically below 80 mV/cm·Oe^17^. Besides variation of the connectivity type and optimization of microstructure, the ME effect can be enhanced by using materials with larger piezoelectric coefficients.

Recently, a very large piezoelectric effect has been discovered in solid solutions of (1−*x*)Ba(Ti_0.8_Zr_0.2_)O_3_-(*x*)(Ba_0.7_Ca_0.3_)TiO_3_ (BZT-*x*BCT)^18^,^19^,^20^,^21^,^22^,^23^,^24^. The BZT-*x*BCT system constitutes a line on the ternary phase diagram between the end members BaTiO_3_, CaTiO_3_, and BaZrO_3_ (see the inset in Fig. 1(a)). Upon increasing BCT content the room temperature ferroelectric crystal structure changes its symmetry from rhombohedral (R-phase) to orthorhombic (O-phase) and then to tetragonal (T-phase)^23^. It has been proposed that the phase diagram of BZT-*x*BCT involves two lines of polymorphic phase boundaries (between the T and O phases and between the O and R phases) with an interleaving two-phase coexistence (R-T phase)^25^ or single phase region (O-phase only)^21^. It was found that values of the piezoelectric coefficient, *d*_33_, along the orthorhombic to tetragonal phase boundary are much higher than that along the orthorhombic to rhombohedral phase boundary^26^. The extraordinary piezoelectric properties were attributed to a reduction in the crystalline anisotropy of polarization, and elastic softening (increase in the elastic compliance constant 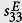)^26^. In particular, for the composition with *x* = 0.5 in BZT-*x*BCT (later BCZT), which lies on the R-T phase boundary, the piezoelectric coefficient *d*_33_ reaches ~600 pC/N at room temperature. The excellent electromechanical properties make BCZT a promising candidate for the development of multiferroic composites with large ME coefficients. However, to our knowledge there have not been reports on multiferroics based on this compound, yet.

In this article we report on the synthesis, multiferroic properties and the magnetoelectric effect for the [BCZT]−[CoFe_2_O_4_] in 85:15 weight% ratio composite ceramics, here onwards referred to as BCZT_85_-CFO_15_. CoFe_2_O_4_ (CFO) is often used as the magnetic constituent in multiferroic composites due to its large magnetostriction^27^.

## Results and Discussion

**Figure 1:**
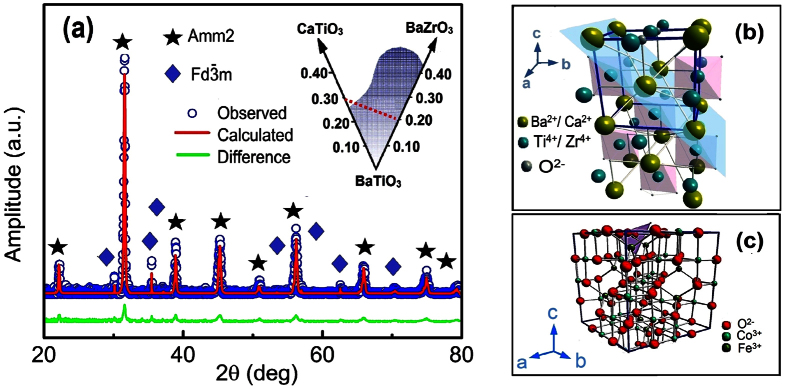
(**a**) The XRD pattern of the BCZT_85_-CFO_15_ ceramics measured at room temperature and the pattern calculated by Rietveld refinement based on the sum of an orthorhombic structure with the space group *Amm*2 shown in (**b**) and cubic inverse spinel structure with space group 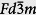 shown in (**c**). The inset in panel (a) shows the ternary phase diagram of BaTiO_3_, CaTiO_3_, and BaZrO_3_ adapted from Ravez *et al.*^24^. The dashed red line represents the full composition BZT-*x*BCT.

The phase content and structure of the BCZT_85_-CFO_15_ ceramics were characterized by X-ray diffraction (XRD). The observed XRD pattern, the calculated pattern from the Rietveld analysis, and the one showing the difference between the two are shown in Fig. 1(a). The observed diffraction peaks can be indexed as the perovskite orthorhombic structure with space group *Amm*2 for BCZT (Fig. 1(b)), as was shown for similar compositions by Acosta *et al.*^23^, and as the cubic spinel structure with space group 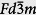 for CFO (Fig. 1(c)). The Rietveld refinement analysis revealed no extra phases and the calculated peak intensities matched well with the observed ones. In the composites, the lattice parameters for the BCZT phase are: *a*_*BCZT*_ = 4.002 Å, *b*_*BCZT*_ = 5.676 Å and *c*_*BCZT*_ = 5.656 Å, while CFO has a lattice parameter of *a*_*CFO*_ = 8.4 Å.

**Figure 2:**
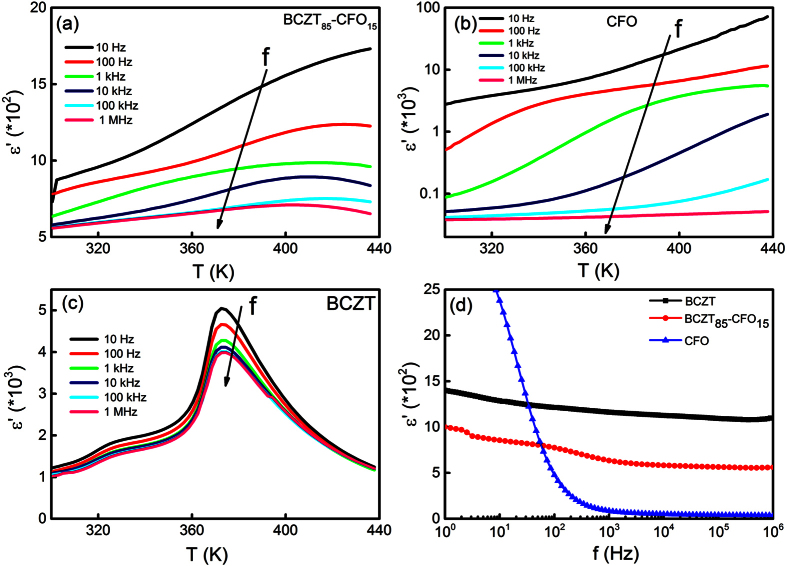
(**a**) Temperature dependence of the real part of the electric permittivity of the BCZT_85_-CFO_15_ composite measured at different frequencies (see Supplementary Information Fig. S1 for *ε′′*(*T*) measured in the frequency range from 10 Hz to 1 MHz). (**b**) Temperature dependence of real part of the electric permittivity for pure CFO measured at different frequencies from 10 Hz to 1 MHz range. (**c**) Temperature dependence of the electric permittivity for single phase BCZT ceramics. (**d**) Frequency dependence of the real part of electric permittivity for CFO, single phase BCZT and the BCZT_85_-CFO_15_ composite measured at 300 K. Arrows point in the direction of increasing frequency.

Figure 2(a,c) show the temperature dependence of the real part of electric permittivity *ε′* for BCZT_85_-CFO_15_ composite and for pure BCZT ceramic, respectively, measured at different frequencies. For BCZT, *ε′(T)* exhibits a maximum at around 370 K, which corresponds to the ferroelectric-paraelectric phase transition, and another anomaly at 320 K related to the polymorphic phase transition between the ferroelectric orthorhombic and tetragonal phases^23^,^26^. The composite sample shows markedly different behavior. A broad peak is observed around 400–420 K for frequencies above 100 Hz. At lower frequencies, the permittivity continuously increases with temperature. No low temperature anomaly can be revealed. This substantially different dielectric behavior of the two samples may be attributed to the contribution of CFO phase that has lower electric permittivity especially at high frequencies. The comparison between electric permittivity of the BCZT, CFO, and the composite at room temperature is illustrated in Fig. 2(d), showing frequency spectra *ε′(f)* for all three samples. Electric permittivity of pure CFO continuously grows with increasing temperature (Fig. 2(b)) and the growth rate becomes strong as frequency decreases. A particular feature of the composite sample is the strong frequency dispersion of the electric permittivity. The position of the broad maxima *ε′*_*m*_ for the composite shifts to higher temperatures with increasing frequency and the same happens for the imaginary part of the electric permittivity (see Supplementary Information Fig. S1). Similar dielectric behavior has been observed in other multiferroic composites containing ferrites e.g. Ni_0.8_Zn_0.2_Fe_2_O_4_-Sr_0.5_Ba_0.5_Nb_2_O_6_^28^, Ba_0.6_Sr_0.4_TiO_3_-Ni_0.8_Zn_0.2_Fe_2_O_4_^29^, and Pb(Zr_0.53_Ti_0.47_)O_3_-(Ni_0.5_Zn_0.5_)Fe_2_O_4_^30^.

**Figure 3:**
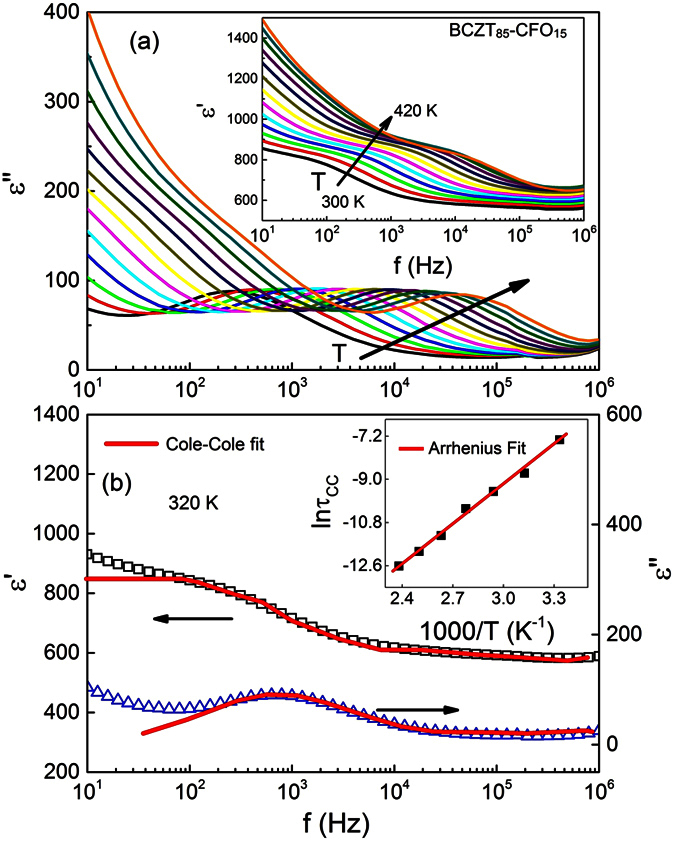
(**a**) *ε′′*(*f*) plots for the BCZT_85_-CFO_15_ composite at different temperatures. The inset shows the corresponding *ε′*(*f*) plots. (**b**) Representative graphs showing Cole-Cole fitting on the real and imaginary part of electric permittivity for the composite. The inset shows the plot between inverse temperature and ln*τ*_*CC*_, where *τ*_*CC*_ corresponds to the Cole-Cole relaxation time obtained from permittivity fitting to Eqs (2) and (3).

To study the dielectric relaxation in more detail, we measured the frequency dependence of the electric permittivity at different temperatures. The isotherms of the imaginary part of the permittivity of the composite are shown in Fig. 3(a), while the isotherms of the real part are shown in the inset. A strong increase of both *ε′* and *ε″* at low frequencies is mainly caused by the contribution from the CFO inclusions, which affects the properties of the composite via two mechanisms. First, due to the intrinsic conductivity of cobalt ferrite, which is activated at low frequencies and higher temperatures. Secondly, due to the interfaces between the more conductive ferrite and more insulating BCZT phase, which gives rise to the Maxwell-Wagner type interfacial relaxation. These mechanisms were also suggested for BaTiO_3_−Ni_0.5_Zn_0.5_Fe_2_O_4_ composites^31^,^32^. Maxwell–Wagner relaxation is usually related to inhomogeneities and interfaces that are more pronounced in grained ceramics and composites^32^. A large number of interfaces between the ferroelectric and ferrimagnetic phases acts as traps for the mobile charge carriers. In the vicinity of the interfaces, the polarization is strongly inhomogeneous giving rise to uncompensated surface/interface charges that lead to the formation of inner fields and cause huge dielectric losses, especially at low frequencies where the separation distances between the positive and negative charges can be substantial^32^.

Besides the low-frequency relaxation, *ε′(f)* and *ε″(f)* show a broad step and a broad maximum in the frequency range 10^2^ < *f* < 10^5^ Hz, respectively, whose positions shift towards higher frequency as temperature increases. This indicates a thermally activated relaxation process. To evaluate the physical nature of these relaxations, we fit the complex permittivity with the Cole-Cole model^33^:


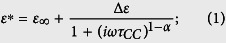


where *ε*_∞_ is the permittivity in the high frequency limit, Δ*ε* = *ε*_*s*_ − *ε*_∞_ where *ε*_*s*_ is the static, low frequency permittivity, *τ*_*CC*_ is the characteristic relaxation time, and ω is the angular frequency.

Equation (1) can be decomposed into real and imaginary parts as:


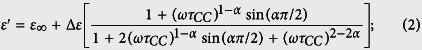


and


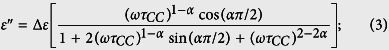


The exponent *α* describes the symmetric broadening of the relaxation time distribution: *α* *=* 0 corresponds to the canonical Debye relaxation (a system having a single relaxation time), while *α* > 0 describes a distribution of the relaxation times. Fittings to eqs (2 and 3) are shown in Fig. 3(b) while the best fit parameters are presented in Table 1. The temperature activated dynamics of the relaxation process can be described by the Arrhenius behavior 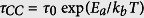 where *τ*_0_ is the pre_*-*_exponential factor, *E*_*a*_ is the activation energy, *k*_*b*_ is the Boltzmann constant, and *T* is absolute temperature. The best fit (solid red line in the inset Fig. 3(b)) yields *E*_*a*_ = 0.47 ± 0.01 eV and *τ*_0_ = (8.4 ±* *0.2) × 10^−12^ s. The value of the activation energy is in accordance with that calculated for pure CFO (see Supplementary Information Fig. S5), literature data available for cobalt ferrite^34^ and other ferrite containing compounds^29^,^32^,^35^ which is attributed to the electronic conduction process between Fe^2+^ and Fe^3+^ ions. As a matter of fact, the ferrite phase when sintered at such high temperature, contains a number of intrinsic oxygen vacancies^36^. For the charge balance in the system, Fe^3+^ transforms to Fe^2+^ to generate electrons to compensate the positive charge at the oxygen vacancy. The weakly bound electrons on Fe^2+^ will transit between the ferrous and ferric ions by hopping under the electric field, a phenomenon known as the Vervey-de Boer mechanism^37^.

**Figure 4:**
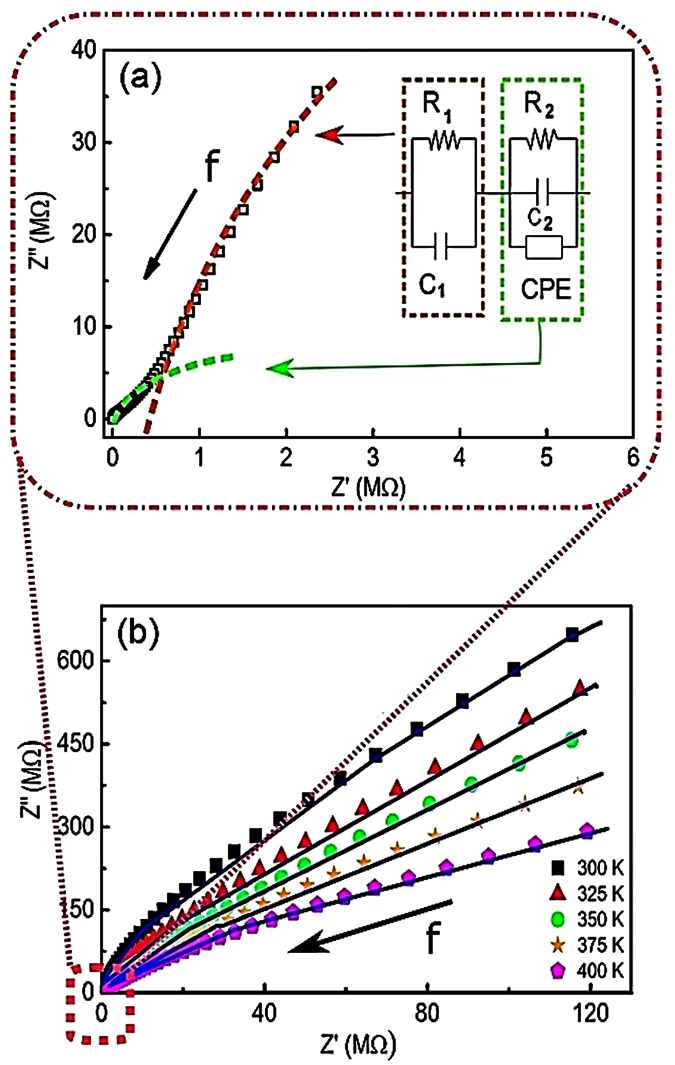
(**a**) Nyquist plot for impedance of the composite at 300 K showing two unresolved semicircles. The inset shows the equivalent circuit used to model the circuit to obtain the electrical parameters. (**b**) Equivalent circuit modeling applied at different temperatures. Symbols correspond to data points whereas the solid lines represent the best fits.

Nyquist (Z′ vs. Z′′) plots, where Z′ and Z′′ stand for the real and imaginary parts of electric impedance, were analyzed to obtain a deeper insight into the relaxation processes. Typically Nyquist plots are modeled by several serially connected equivalent electrical circuits, corresponding to different relaxation processes. For our composite sample we have expected a superposition of contributions related to the perovskite grains, the ferrite grains and interfaces between them. From the Nyquist plot for the BCZT_85_-CFO_15_ ceramic at 300 K, we can see two poorly resolved semicircular arcs as illustrated in Fig. 4(a). Therefore two equivalent circuits were used for the fitting (see the inset of Fig. 4(a)). Here *C*_1_ and *C*_2_ are the capacitances, *R*_1_ and *R*_2_ correspond to resistances, and CPE is a constant phase element representing the non-ideal dielectric response correlating the *ac* conductivity with the movement of charge carriers inside the grains. The impedance of the CPE is given by 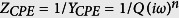, where *Q* is the pre-factor whose value is proportional to the capacity of the sample, and the value of the exponent *n* varies from 0 (for a purely resistive element) to 1 (for a purely capacitive one)^38^. The temperature evolution of these plots is shown in Fig. 4(b) and the best fitting parameters are summarized in Table 2. We have compared the obtained parameters with results of the equivalent circuit fitting of Nyquist plots of the pure BCZT and CFO sample presented in the Supplementary Information (Figs S2 and, S3 and Table S1). We found that the second circuit shown in Fig. 4(a) has parameters pretty close to that of the pure ferrite sample, e.g. the resistance is of the order of 10^6^ ohms while Q is of the order of 10^−10^ *S.*s^*n*^ at 300 K (see Table S1 in Supplementary Information). Therefore we can attribute it to the contribution of ferrite phase. At the same time, we could not resolve contributions of perovskite phase and interfaces, therefore we assume that the first circuit represents an effective response of perovskite grains and interfaces.

The value of CPE exponent decreases with increasing temperature showing increase in non-ideality. Here we want to emphasize the fact that although the equivalent circuit modelling is used to reveal the dielectric relaxation mechanisms in composite ceramics, the overall permittivity is influenced by the conductivity inhomogeneity and the presence of electric charge. The conductivity inhomogeneity is a result of the large difference of conductivities of ferrite and the perovskite phase, which can cause changes in the conductivities of these individual phases. Hence the impedance analysis, although it appears complete, is still an approximation as compared to the actual relaxation processes in such complex systems. On the one hand, the Cole-Cole equation (Eq. 1) is used to describe the distribution of relaxation times while discussing the electric permittivity data. On the other hand, CPE is used in equivalent circuit analysis. Macdonald^39^ pointed out that the parameter *n* of CPE and the exponent α of the Cole-Cole relaxation are related to each other via a relationship *n* *=* 1*−α,* i.e. the values estimated from fitting with these forms should be comparable. Indeed, by comparison of the parameters presented in Table 1 and Table 2, one can see that 1−α and *n* values are comparable in the temperature range 350–400 K.

**Figure 5:**
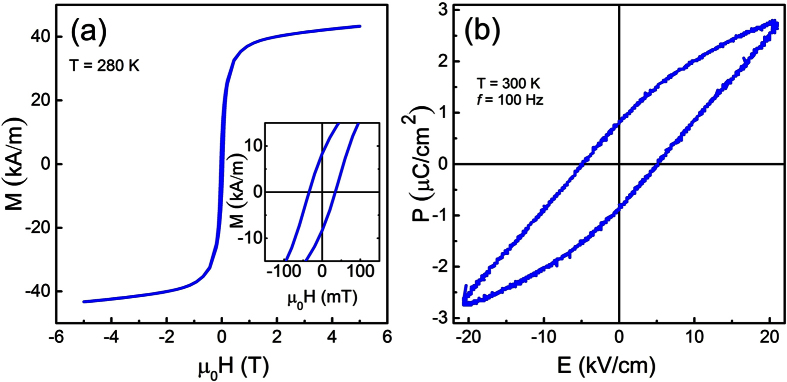
(**a**) Magnetization (*M*) versus dc magnetic field (*μ*_*0*_*H*) curve. The inset shows a zoom-in image of the same curve to show remnant magnetization and coercive field. (**b**) Polarization vs. electric field hysteresis loop measured for the BCZT_85_-CFO_15_ composite at room temperature (300 K).

We, from here onwards, characterize the composite for multiferroic and magnetoelectric properties. The room temperature magnetic field dependence of the magnetization, (*M*-*H*) loop, was measured via superconducting quantum interference device (SQUID) magnetometry (MPMS-5S, Quantum Design). The curve shown in Fig. 5(a) exhibits a very slim hysteresis loop with remanent magnetization, 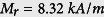, and relatively small coercive field, *μ*_*0*_*H* = 0.04 T, as illustrated in the inset, confirming the ferrimagnetic state of the sample. The sample exhibits a ferroelectric-like hysteresis (*P*-*E*) loop (Fig. 5(b)). The values of remnant polarization and coercive field are 0.9 *μ*C/*cm*^2^ and 5 *kV*/*cm*, respectively. Similar to other multiferroic bulk composites, the polarization hysteresis loop is not fully saturated and shows a contribution from leakage current^40^.

**Figure 6:**
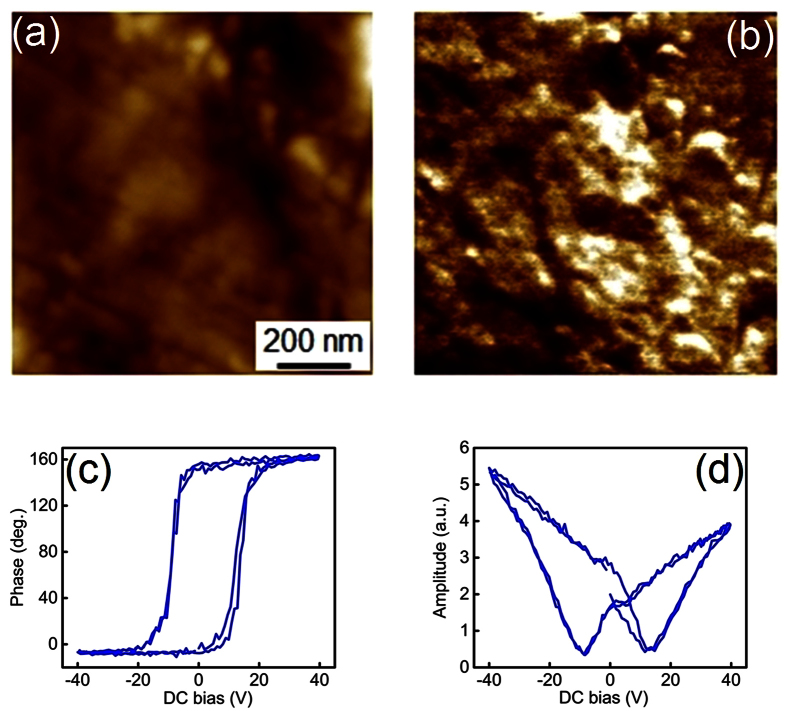
(**a**) Room temperature topography image of the polished sample surface. (**b**) Lateral piezoresponse force microscopy image of the same area as in (**a**). The bright regions display piezo-electrically active phase. (**c**) Local piezoresponse force microscopy phase loop and (**d**) Amplitude hysteresis loop measured inside a piezoactive region.

To measure the microscopic piezoelectricity of the composite we conducted piezoresponse force microscopy (PFM) studies. Figure 6(a,b) show the topography and lateral PFM images, respectively, of the polished sample surface, acquired at room temperature. Regions of the distinct contrast in Fig. 6(b) correspond to the piezoactive BCZT phase. The PFM contrast, i.e. the local polarization, can be reversed by applying a *dc* voltage above a certain threshold value, signifying the ferroelectric state of BCZT. Figure 6(c,d) show typical local switching spectroscopy PFM loops (phase and amplitude respectively). As can be seen, the phase of the PFM signal exhibits nearly ideal 180° switching which is typical of ferroelectric materials. Thus, both macroscopic and microscopic measurements confirm that in the composite sample the CFO and BCZT phases retain their ferrimagnetic and ferroelectric character i.e. our composite is an extrinsic multiferroic. To address the coupling between magnetic and electric degrees of freedom we measured the converse magnetoelectric effect, *M*_*ME*_(*E*).

**Figure 7:**
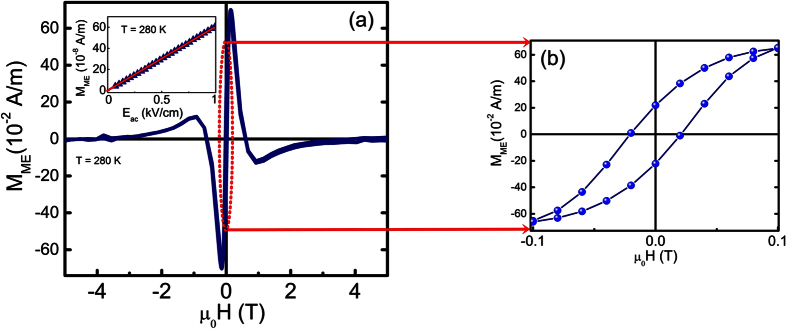
(**a**) Magnetoelectrically induced magnetization, *M*_*ME*_, as a function of superimposed dc magnetic field for the BCZT_85_-CFO_15_ composite ceramics. The inset shows *M*_*ME*_ vs. applied ac electric field (*μ*_*0*_*H*_*dc*_ = 0.2 T), the solid line corresponds to the best fit to the linear dependence. From the slope of the line the converse magnetoelectric coefficient was calculated. The zoomed-in figure (**b**) shows the hysteresis for *M*_*ME*_ at low magnetic fields. Standard deviations in the experimental data are of the order of the line thickness and the symbols size used.

The magnetoelectric measurements were performed using a modified SQUID ac-susceptometer^41^. In this method, the *ac* magnetic moment induced by an *ac* electric field applied to the sample is measured. The inset in Fig. 7(a) shows the magnitude of the induced magnetization measured at room temperature as a function of the *ac* electric field amplitude. The *M*_*ME*_(*E*_*ac*_) dependence is linear and hysteresis free. The best linear fit yields the value of the converse ME coefficient, *α*_*CME*_ = (6.03 ± 0.03) ps/m. This value is about 50% larger than the ME coefficient of the bulk multiferroic composite of [BaTiO_3_]_0.8_-[CoFe_2_O_4_]_0.2_ produced via normal sintering *α*_*CME*_ ~ 4 ps/m^42^, although containing a higher amount of CFO, namely 20 weight percent. It is known, that in BaTiO_3_-CoFe_2_O_4_ composites with increasing CFO content the magnetoelectric effect first increases, reaches a maximal value at 40–50 weight percent of CFO, and then decreases again^43^. Therefore, the larger ME effect observed in our samples in spite of the lower CFO content, can be ascribed to the larger piezoelectric coefficient of the BCZT material as compared to BaTiO_3_. One can expect that even stronger magnetoelectric coupling can be achieved in materials with larger content of the ferrite phase. However, usually increasing CFO content results in decreasing resistivity of the composites, which can drastically deteriorate the measurable magnetoelectric effect. Presently, we carry out efforts to sinter composite ceramics with larger CFO content, keeping the resistivity in check, to get the maximum possible ME values which will beat, in our view, the values obtained for BTO-CFO composites. These efforts have been partially successful and will be discussed in a separate report.

Figure 7(a) shows the ME induced magnetization, *M*_*ME*_, at given *ac* electric field (1 kV/cm) as a function of a superimposed *dc* magnetic field while Fig. 7(b) shows its zoomed-in view at lower field values. The shape of the *M*_*ME*_(*H*) curve is typical for bulk multiferroic composites containing CFO^44^,^45^. Indeed, since the ME effect in such composites is due to strain coupling at the interface, the magnetoelectric coefficient can be written as 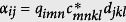, where *q*_*imn*_ is the piezomagnetic coefficient, 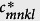 is an effective stiffness of the microstructure, and *d*_*jkl*_ is the piezoelectric coefficient^43^. For our experimental conditions, the magnetic field and measured magnetic moment are perpendicular to the electroded sample faces, the shape of the *M*_*ME*_(*H*) curve should follow the magnetic field dependence of the longitudinal piezomagnetic coefficient, *q*_*l*_, that in turn is determined by the field dependence of magnetostriction *λ*, *q*_*l*_ = *dλ*/*dH* (here the effective uniaxial value of a sample). For polycrystalline CoFe_2_O_4_, the *λ*(*H*) dependence is non-monotonous exhibiting a maximum at *μ*_0_*H* = 0.3–0.4 T^46^,^47^. Therefore, *q*_*l*_ changes sign at the field corresponding to the maximal magnetostriction, resulting in the sign reversal of the ME response. Finally, at very large magnetic field, magnetostriction saturates and the magnetoelectric coefficient tends to zero. The maximal ME effect is reached at the magnetic field of *μ*_0_*H* = 0.2 T that corresponds to the maximal *dλ/dH.* This field is larger than for the [BaTiO_3_]_0.8_-[CFO]_0.2_ composite (*μ*_0_*H* = 0.15 T)^42^. The difference might be related to the different grain size of CFO.

## Conclusions

In conclusion, we have successfully prepared the bi-phasic piezoelectric-ferrimagnetic composite of BCZT_85_-CFO_15_ via the solid state reaction method. The dielectric response of the composite is partly affected by the contribution from the ferrite phase. The low frequency dielectric relaxation is attributed to Maxwell-Wagner relaxation. Analysis of ferroelectric and ferrimagnetic properties at room temperature proves the multiferroic character of the composite. The obtained data allow to consider BCZT_85_-CFO_15_ composite ceramics as promising for the development of lead free materials with larger magnetoelectric coupling at room temperature. Even a relatively small amount of ferrimagnetic phase (15%) resulted in a sizable magnetoelectric coupling, the magnetoelectric coefficient reaches 6.03 ps/m. When comparing to reported values from the literature, the values of the piezoelectric coefficients of BCZT (*d*_33_ = 600 pC/N)^18^ and BaTiO_3_ (*d*_33_ = 191 pC/N)^16^, one can expect even larger ME coupling in BCZT based composites. It means that there are other factors besides the intrinsic piezoelectric coefficient and the ratio between the ferroelectric and ferrimagnetic phase, which affect the ME effect. An important role is played by microstructure: grain size, density of ceramics, and quality of interfaces. Another one can be the difference between the stiffness parameters of BCZT and CFO. The exact value of stiffness parameter of BCZT is so far unknown. Control of these parameters should improve the magnetoelectric coupling in the studied material.

## Methods

The BCZT powder was prepared employing a two-step solid state reaction using reagent grade carbonates and oxides. The raw powders CaCO_3_ (Fluka Chemie GmbH, purity > 99%)), BaCO_3_ (Alfa Aesar GmbH KG, purity 99.95%), TiO_2_ (Merck KgaA, purity > 99%) and ZrO_2_ (Sigma Aldrich U.K. purity 99%) were mixed according to the stoichiometric formula 0.5Ba(Zr_0.2_Ti_0.8_)O_3_−0.5(Ba_0.7_Ca_0.3_)TiO_3_. This composition was chosen keeping in mind the phase diagram of BZT-*x*BCT^19^,^23^ with special emphasis on the high piezoelectric constants of this composition. All the powders were added to an alumina milling container with yttria stabilized zirconia balls and ethanol. The suspensions was ball-milled in a planetary ball mill (Fritsch Pulverisette 5) for 12 hours at 250 rpm and dried at room temperature for 24 h. The obtained powder was calcined at 1400 °C for 6 h with a heating rate of 5 °C/min and subsequently ball-milled for 10 hours with the previously described parameters. The powder was again calcined for another 6 hours at 1500 °C at a rate 5 °C/min and ball milled subsequently for 10 hours as previously.

CoFe_2_O_4_ was also prepared by the mixed oxide route. The oxides of iron and cobalt i.e. Fe_2_O_3_ (Alfa Aesar GmbH KG, purity > 99%) and Co_3_O_4_ (Alfa Aesar GmbH KG, purity > 99%) were mixed together in ethanol in stoichiometric ratios and milled for 10 hours in alumina crucible with yttria stabilized zirconia balls for 10 hours. The suspension was dried and calcined for 6 hours at 1050 °C with a heating rate of 5 °C/min and ball milled again with the previously described parameters. The calcined powders BCZT and CFO were mixed together in 85% and 15% weight % ratio in ethanol and milled for 6 hours at 300 rpm. The suspension was dried and the obtained powder was pressed into disk shaped pellets of 6 mm diameter and 1 mm thickness using a hydraulic press under uniaxial pressure of 300 MPa for 2 min. The pellets were sintered in covered alumina crucibles in air at 1300 °C for 6 hours and with the heating rate of 5 °C/min.

X-ray diffraction was performed at room temperature using a Siemens D5000 diffractometer both for the calcined powders and for the final sintered pellets. Rietveld refinement was performed using Highscore plus software^48^. The structures shown in Fig. 1(b,c) were drawn with Diamond-Crystal Structure and visualization software^49^. For piezoresponse force microscopy studies the samples were polished with fine polishing papers up to 1 micron or 8000 grit. Measurements were performed using a commercial scanning probe microscope (MFP-3D, Asylum Research). For electrical measurements, the sintered pellets were ground to 900 μm thickness and silver electrodes were fired at 500 °C on both faces. Macroscopic polarization versus electric field (P-E) loops were studied using a custom-made Sawyer–Tower circuit at an electric field frequency of 100 Hz. Dielectric measurements were accomplished using a Solartron 1260 impedance analyzer with the dielectric interface 1296. The magnetic measurements were performed by SQUID magnetometry (MPMS-5S, Quantum Design). The converse magnetoelectric effect was measured using a modified ac susceptometer of the SQUID magnetometer, where the first harmonic of the *ac* magnetic moment induced by an external *ac* electric field (*f*_*ac*_  = 11 Hz) is measured^41^. We addressed the longitudinal magnetoelectric effect, where the applied magnetic field, applied electric field, and the measured magnetization are parallel to each other and perpendicular to the sample surface.

## Additional Information

**How to cite this article**: Naveed-Ul-Haq, M. *et al.* A new (Ba, Ca)(Ti, Zr)O_3_ based multiferroic composite with large magnetoelectric effect. *Sci. Rep.* **6**, 32164; doi: 10.1038/srep32164 (2016).

## Supplementary Material

Supplementary Information

## Figures and Tables

**Table 1:** Summary of fitting results according to Cole-Cole equations (equations 2 and 3).

*T* (K)	*τ*_*CC*_ (*μs*)	*Δε*	*α*
300	637 ± 14	307 ± 5	0.33 ± 0.01
320	159 ± 10	301 ± 4	0.32 ± 0.01
340	74 ± 7	298 ± 5	0.31 ± 0.01
360	36 ± 4	278 ± 7	0.29 ± 0.02
380	12 ± 4	263 ± 4	0.26 ± 0.01
400	6.0 ± 0.4	252 ± 10	0.22 ± 0.01
420	3.0 ± 0.5	217 ± 11	0.16 ± 0.03

**Table 2:** Best fit parameters according to the circuit Fig. 4(a).

*T* (K)	*C*_1_ (nF)	*C*_2_ (pF)	*R*_1_ (GΩ)	*R*_2_ (MΩ)	*Q* (*10^−10^S·s^n^)	*n*
300	18.7 ± 0.6	160 ± 10	6.20 ± 0.80	2.2 ± 0.3	1.02 ± 0.01	0.80 ± 0.10
325	12.6 ± 0.4	250 ± 20	0.93 ± 0.03	1.6 ± 0.2	1.42 ± 0.05	0.80 ± 0.01
350	16.7 ± 1.0	310 ± 15	0.13 ± 0.01	0.45 ± 0.05	2.10 ± 0.04	0.78 ± 0.03
375	15.1 ± 1.0	570 ± 20	0.12 ± 0.02	0.11 ± 0.01	2.00 ± 0.01	0.75 ± 0.02
400	11.5 ± 0.6	340 ± 20	0.09 ± 0.01	0.13 ± 0.04	1.04 ± 0.07	0.74 ± 0.01
