# Supplementary material for: A new (Ba, Ca) (Ti, Zr)O3 based multiferroic composite with large magnetoelectric effect
Source: Sci Rep. 2016 Aug 24;6:32164. doi: 10.1038/srep32164 (PMC4995375; doi:10.1038/srep32164)
Supplement: Supplementary Information [file srep32164-s1.pdf]

On-line Supplementary Information for

A new (Ba, Ca)(Ti, Zr)O_3 based multiferroic composite with large magnetoelectric effect

M. Naveed-Ul-Haq*[, 1], Vladimir V. Shvartsman[1], Soma Salamon[2], Heiko Wende[2], Harsh Trivedi[1], Arif Mumtaz[3], Doru C. Lupascu[1]

[1] Institute for Materials Science and Center for Nanointegration Duisburg-Essen (CENIDE), University of Duisburg-Essen, Universitätsstraße 15, 45141 Essen, Germany.

[2] Faculty of Physics and Center for Nanointegration Duisburg-Essen (CENIDE), University of Duisburg-Essen, Lotharstraße 1, 47057 Duisburg, Germany.

[3] Department of Physics, Quaid-i-Azam University, Islamabad 45320, Pakistan.

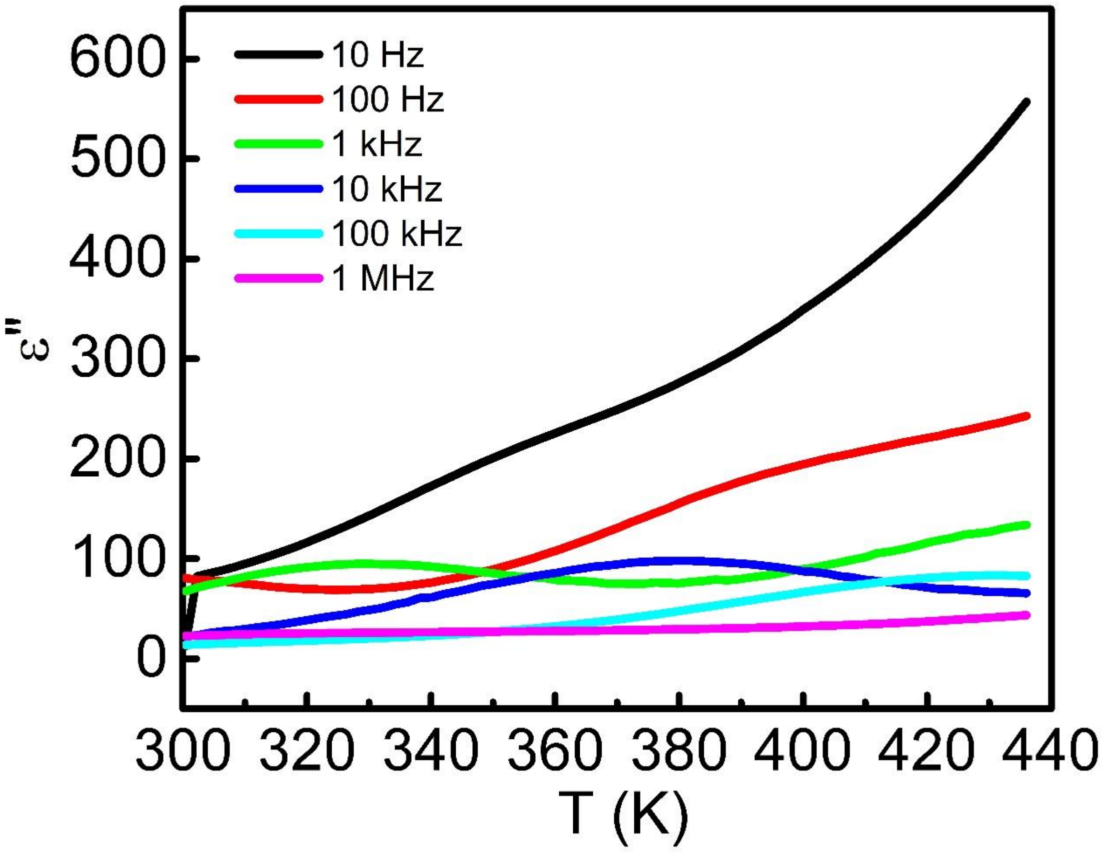

Fig. S1. Imaginary part of dielectric permittivity as a function of temperature for the composite $BCZT_{85}$-CFO_{15} measured in the frequency range 10 Hz - 1 MHz.

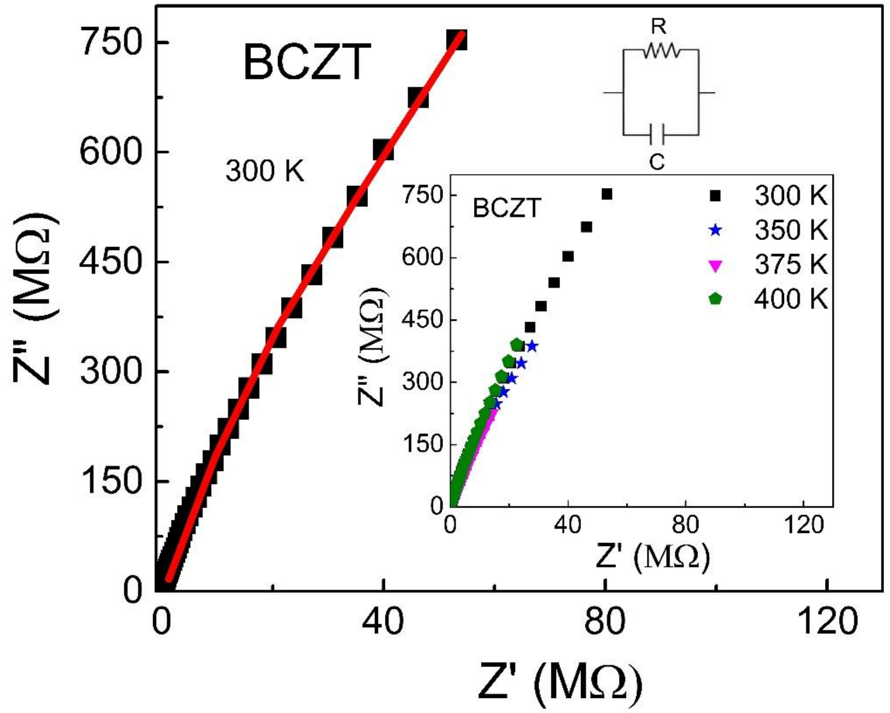

Fig. S2. Nyquist plot for BCZT fitted to the circuit shown. The symbols are the experimental data while the lines correspond to the fitting. Parameters values are: R = $(3.9 \pm 0.3) \times 10^{9}$ Ω and C = $(1.2 \pm 0.2) \times 10^{-12}$ F. The inset shows the Nyquist plots for BCZT at different temperatures.

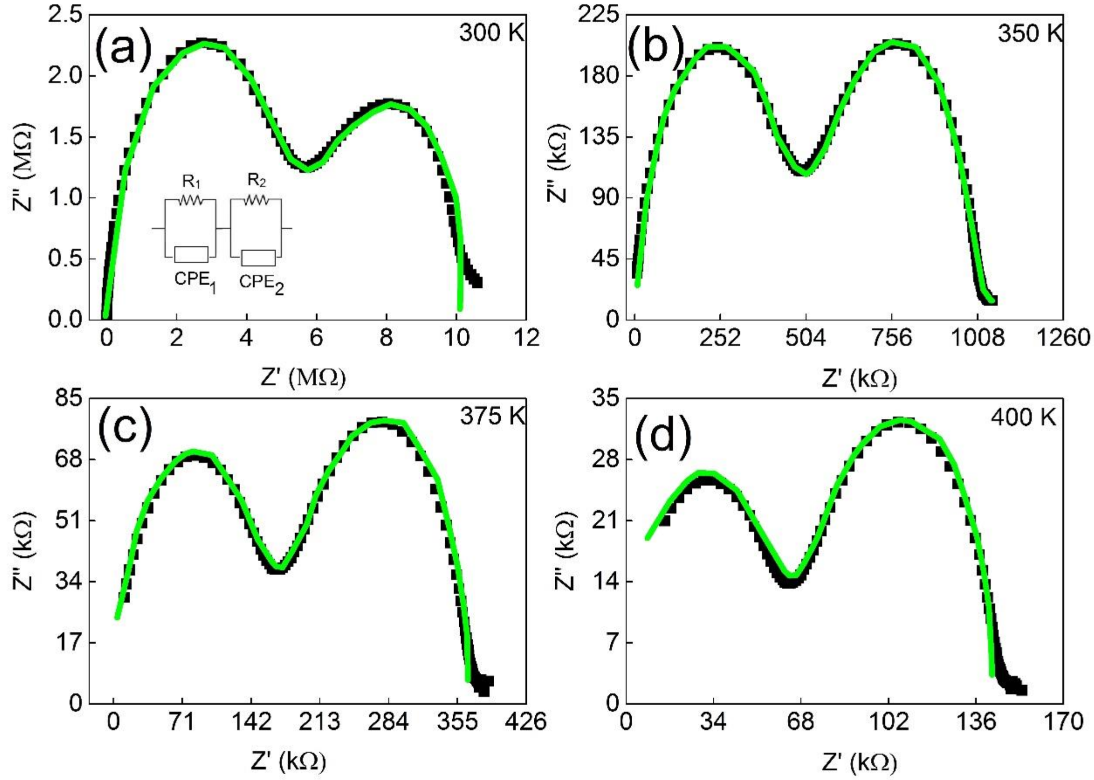

Fig. S3. Nyquist plot for CFO at different temperatures fitted to the circuit shown in (a). The symbols are the experimental data while the solid lines correspond to the fitting. The parameters are summarized in table S1.

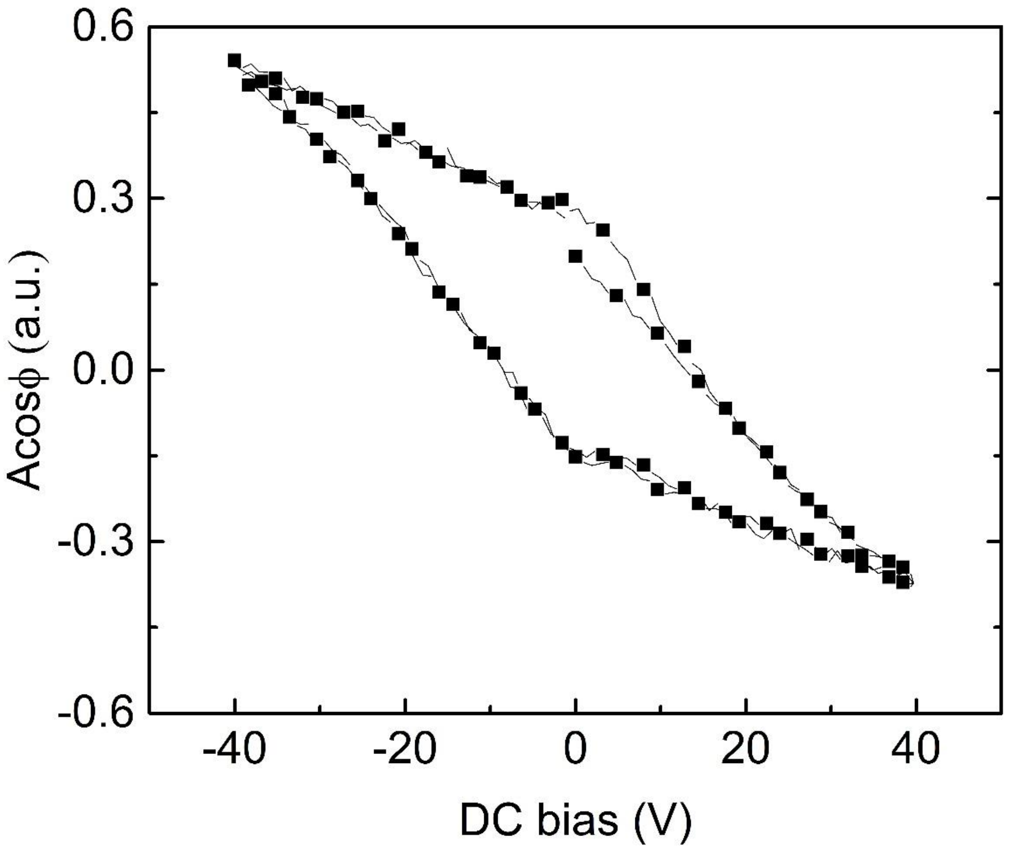

Fig. S4. Piezoresponse of the composite calculated from the amplitude and the phase shown in Fig. 6 (c) and (d) of the manuscript.

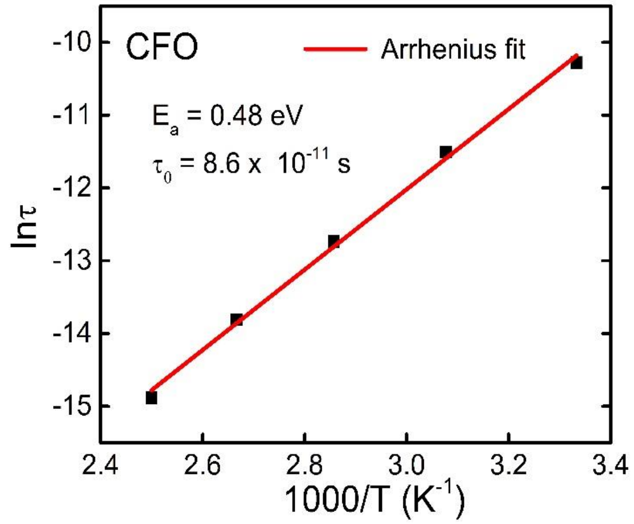

Fig. S5. Arrhenius fit to pure CFO data. The values of activation energy and characteristic time are of the same order as those for the composite mentioned in the manuscript text. Errors in fitting results are less than 1 % of the values shown on the graph.

Table S1: Fitting results to the Nyquist plots (Fig. S3 (a-d)) according to circuit in Fig. S3 (a). Errors are less than 1% of the values.

Temperature	R_1 (Ω)	Q_1 (S·s^n)	n_1	R_2 (Ω)	Q_2 (S·s^n)	n_2
300 K	5.1×10^{6}	1.2×10^{-10}	0.88	5.6×10^{6}	1.5×10^{-9}	0.68
350 K	5.8×10^{5}	7.4×10^{-9}	0.73	4.5×10^{5}	2.2×10^{-11}	0.90
375 K	1.6×10^{5}	3.3×10^{-11}	0.87	2.1×10^{5}	8.3×10^{-9}	0.76
400 K	8.6×10^{4}	9.2×10^{-9}	0.78	6.2×10^{4}	5.6×10^{-11}	0.84
